# Developmental ability of oocytes retrieved from Meishan neonatal ovarian tissue grafted into nude mice

**DOI:** 10.1111/asj.13160

**Published:** 2019-01-17

**Authors:** Hiroyuki Kaneko, Kazuhiro Kikuchi, Nguyen Thi Men, Junko Noguchi

**Affiliations:** ^1^ Institute of Agrobiological Sciences National Agriculture and Food Research Organization (NARO) Tsukuba Ibaraki Japan; ^2^ The United Graduate School of Veterinary Science Yamaguchi University Yamaguchi Japan

**Keywords:** developmental ability, follicular growth, Meishan pigs, oocyte growth, ovarian xenografting

## Abstract

Ovarian xenografting makes it possible to obtain oocytes with fertilization ability from immature pigs of Western breeds. In this study, we applied these methods to the Meishan, an indigenous Chinese pig breed, and investigated the developmental competence of oocytes grown in their neonatal tissue after grafting into nude mice. First, mice harboring neonatal ovarian tissue were infused with follicle stimulating hormone (FSH) (62.5 U/ml) for 13 days starting at 10, 30, and 60 days after vaginal opening (D10‐, D30‐, and D60‐FSH groups, respectively). Development of antral follicles and their oocytes was most enhanced in the D60‐FSH group. For the next step, we examined the in vitro maturation ability of the oocytes recovered from host mice after infusion with FSH at a dose of 62.5 U/ml or 125 U/ml (FSH‐62.5 or ‐125 group) for 13 days starting at 60 days after vaginal opening. Many more oocytes with maturation ability were obtained from the FSH‐125 group. The FSH‐125 mature oocytes were fertilized in vitro, as shown by formation of male and female pronuclei, but did not reach the blastocyst stage. These results indicate that Meishan neonatal ovaries are able to produce oocytes with fertilization ability after being grafted into nude mice.

## 1 INTRODUCTION

The neonatal ovary contains a large pool of small and immature oocytes, and could be a potential genetic resource for agricultural purposes if these oocytes could be appropriately grown and given developmental competence. Therefore, techniques for the utilization of immature oocytes have been required. Transplantation of fetal or neonatal ovarian tissue from different donor species into immunodeficient mice (ovarian xenografting) can confer developmental competence to immature oocytes (Kikuchi et al., 2016; Paris & Schlatt, 2007). Immature oocytes of domestic animals grown in host mice acquire maturation ability (pig—Kagawa, Kuwayama, Miyano, & Manabe, 2005; Kaneko, Kikuchi, & Noguchi, 2016; Kaneko, Kikuchi, Noguchi, Hosoe, & Akita, 2003; Kaneko et al., 2006; Kaneko, Nakai, Tanihara, Noguchi, & Kikuchi, 2013; and cattle—Senbon, Ishii, Fukumi, & Miyano, 2005) and fertilization ability (pig—Kaneko et al., 2003, 2006, 2013, 2016; and cattle—Senbon et al., 2005) in vitro.

Indigenous pigs possess unique genetic diversity, unlike commercial pigs of Western origin such as the Landrace, Large White, and Duroc (defined as Western breeds), since they have been adapted to geographically isolated environments for a very long time. Therefore, utilization of germplasm from indigenous pigs would be expected to confer resistance to infectious diseases or climate change in existing commercial breeds. However, according to a report from the Food and Agriculture Organization of the United Nations (FAO), only 15% of pig breeds are free from a risk for continuance (Baumung & Wieczorek, 2015), suggesting that conservation of indigenous pigs using assisted reproductive techniques is important and urgent. The Meishan pig is a Chinese indigenous breed noted for its early sexual maturity and high prolificacy. Meishan gilts develop antral follicles in the ovarian cortex by 2 months of age (McCoard, Wise, & Ford, 2003; Miyano, Akamatsu, Kato, Nanjo, & Kanda, 1990), then exhibit the first estrus at 3–4 months of age (Bazer, Thatcher, Martinat‐Botte, & Terqui, 1988; Faillace, Biggs, & Hunter, 1994; White, McLaren, Dziuk, & Wheeler, 1993), whereas in Western breed gilts antral follicles appear by 3 months of age (Oxender, Colenbrander, van deWiel, & Wensing, 1979) and the first estrous occurs between 6 and 8 months of age (Bazer et al., 1988; Faillace et al., 1994; White et al., 1993). Comparative studies of litter size in Meishan and Western breeds have indicated that the Meishan pig delivers three or more piglets (Haley & Lee, 1993). Thus, reproductive parameters in female indigenous pigs are not always similar to those in Western breeds, and few studies of ovarian transplantation using indigenous female pigs have been reported.

In this study, as an example of an indigenous pig breed, we examined the growth of follicles in neonatal ovarian tissue from Meishan pigs that had been transplanted under the kidney capsules of nude mice. We then assessed the developmental competence of the recovered oocytes using in vitro maturation, fertilization, and embryo culture systems.

## 2 MATERIALS AND METHODS

### 2.1 Experimental animals

All experiments were performed in accordance with experimental protocols that were approved by the Animal Care Committee (# H18‐008‐02) of the Institute of Agrobiological Sciences, National Agriculture and Food Research Organization (NARO), Tsukuba, Japan. Meishan pigs used in this study were produced and reared according to the Japanese Feeding Standard for Swine at the Institute of Livestock and Grassland Science of NARO. Female nude mice (Crlj:CD1‐*Foxn1* ^*nu*^), purchased from Charles River Japan (Yokohama, Japan), were kept in an environmentally controlled room maintained at a temperature at 24°C and 70% humidity, and illuminated daily from 05:00 to 19:00 hr.

### 2.2 Chemicals

All chemicals were purchased from the Sigma‐Aldrich Corporation (St. Louis, MO, USA), unless otherwise indicated.

### 2.3 Xenografting of ovarian tissue

Nineteen female Meishan piglets aged 7–14 days were used as donor pigs for experiments 1 (*n* = 6), 2 (*n* = 7), and 3 (*n* = 6). Ovaries were collected, and their cortices were cut into small pieces measuring approximately 1.5 × 1.5 × 1.5 mm in saline supplemented with 660 units/ml penicillin and 0.2 mg/ml streptomycin sulfate (Kaneko et al., 2003, 2006, 2013, 2016). As recipients, 48 female nude mice aged 5−6 weeks were assigned to experiments 1 (*n* = 19), 2 (*n* = 16), and 3 (*n* = 13). All mice in each experiment were anesthetized with pentobarbital sodium (Somnopentyl; Kyoritsu Pharmaceuticals, Tokyo, Japan) followed by isoflurane (Isoflurane for animals; Intervet, Tokyo, Japan) and then ovariectomized. A small hole was made in the mouse kidney capsule with a pair of fine forceps, and approximately 10 fragments of Meishan ovarian tissue were inserted under the capsule of each kidney as described previously (Kaneko et al., 2003, 2006, 2013, 2016). To monitor the formation of antral follicles in the xenografts, vaginal opening in the host mice was observed every 3 days from 20 days after grafting: host mice that had exhibited vaginal opening were used in the following experiments.

### 2.4 Experiment 1

Treatment of host mice with gonadotropins accelerated follicular growth in the Western‐breed grafts and thereby improved developmental competence of the oocytes (Kaneko et al., 2003, 2006). However, there are no available reports on the transplantation of Meishan ovarian tissue into immunodeficient mice. In this study, we first investigated the optimum timing of gonadotropin administration to the host mice to improve follicular growth in the Meishan grafted tissue. Our previous studies using Western‐breed ovaries had indicated that the degree of antral follicle growth in xenografts was affected by the length of time between vaginal opening and gonadotropin treatment of mice (Kaneko et al., 2003), and that infusion of host mice with porcine follicle stimulating hormone (FSH) solution (62.5 U/ml saline) for 14 days was more effective for promoting antral follicle formation than treatment with equine chorionic gonadotropin (eCG) for 2 to 3 days (Kaneko et al., 2006). Eighteen host mice harboring Meishan ovarian tissue were randomly assigned to the following experimental groups: D10 (*n* = 3), D30 (*n* = 3), D60 (*n* = 3), D10‐FSH (*n* = 3), D30‐FSH (*n* = 3), and D60‐FSH (*n* = 3). Mice in the D10‐, D30‐, and D60‐FSH groups were implanted with one osmotic pump (model 2004, DURECT Corp, Cupertino, CA) containing porcine FSH (F2293, 62.5 U/ml saline) for 13 days on days 10, 30, or 60 after vaginal opening (day 0 = vaginal opening), respectively, and then the grafts were recovered. One week after pump implantation, the mice were given an intraperitoneal injection of 100 μl estradiol antiserum raised in a goat (Kaneko et al., 1995, 2002) to inhibit formation of hemorrhagic follicles (Kaneko et al., 2006, 2013, 2016). Mice in the D10, 30, and 60 groups (control groups) received no hormone treatment and their grafts were recovered on days 10, 30, or 60, respectively, after vaginal opening. Ovarian tissue grown under the right kidney capsule in each mouse was subjected to collection of cumulus‐oocyte complexes (COCs), and then the number of recovered COCs was counted. Tissue under the left kidney capsules was used for histological examination. To examine ovarian components before grafting, left ovaries were obtained from three Meishan piglets aged 9 to 14 days, which were different from the ovarian donors.

### 2.5 Experiment 2

The results in experiment 1 indicated that growth of antral follicles and their oocytes was most enhanced in the D60‐FSH group (see Section 3). To examine the dose‐dependent effects of FSH, 12 host mice that had received Meishan ovarian tissue were implanted with one osmotic pump containing either 62.5 or 125 U/ml for 13 days on day 60 with a single injection of estradiol antiserum 7 days after the start of FSH infusion (FSH‐62.5 (*n* = 6) and FSH‐125 groups (*n* = 6), respectively). COCs were recovered from each mouse, then the ability of the recovered oocytes to achieve in vitro maturation were assessed.

### 2.6 Experiment 3

The results of experiment 2 indicated that many more oocytes with maturation ability were obtained from mice in the FSH‐125 group than from those in the FSH‐62.5 group (see Section 3). Thirteen host mice that had received Meishan ovarian tissue were implanted with one osmotic pump containing 125 U/ml FSH for 13 days on day 60 with a single injection of estradiol antiserum 7 days after the start of FSH infusion. COCs were obtained from five mice, then the oocytes were cultured to assess fertilization ability. Oocytes recovered from the remaining mice (*n* = 8) were fertilized in vitro, then cultured further for 6 days to evaluate their ability to reach the blastocyst stage.

### 2.7 Recovery of grafts and oocytes

Host mice in each group were killed by cervical dislocation under anesthesia with isoflurane. COCs were isolated mechanically with a surgical blade in Medium 199 (with Hanks’ salts) (Kikuchi et al., 2002) from antral follicles in tissue grafted under the kidney capsule. In Western‐breed gilts, it had been reported that oocytes larger than 115 μm in diameter acquired in vitro meiotic competence (Hirao et al., 1995; Motlik, Crozet, & Fulka, 1984). On the other hand, Meishan oocytes obtained from antral follicles greater than 4 mm in diameter have been proven to mature in vitro (Xu, Faillace, Harding, Foxcroft, & Hunter, 1998), but the precise relationship between the diameter of oocytes and their developmental ability has not been examined. In this study, therefore, recovered COCs were expediently divided into two groups according to the diameter of the oocytes (<115 μm or ≥115 μm). Only COCs containing oocytes ≥115 μm in diameter were transferred to in vitro maturation.

For histological examination, because the individual grafts were fused together under the kidney capsules in all mice, the entire grafted tissue was excised from the left kidney and fixed in Bouin's solution.

### 2.8 In vitro maturation (IVM), in vitro fertilization (IVF), and in vitro culture (IVC) of oocytes

Recovered COCs containing large oocytes (≥115 μm in diameter) were matured in vitro in North Carolina State University‐37 (NCSU‐37) solution (Petters & Wells, 1993) with modifications (Kikuchi et al., 2002). After IVM, oocytes that had extruded the first polar body were harvested as mature oocytes (at the metaphase II stage) and placed in a modified pig fertilization medium (Suzuki et al., 2002). Frozen spermatozoa prepared from the epididymides of a Meishan boar (Kikuchi et al., 1998) were thawed and incubated in modified Medium 199 (with Eagle's salts; Gibco, Thermo Fisher Scientific, MA) (Nagai et al., 1988), and then a portion of the spermatozoa suspension was introduced into the fertilization medium containing the mature oocytes. The final sperm concentration was adjusted to 1 × 10^5^/ml. For evaluation of fertilization, oocytes were stripped of cumulus cells and attached spermatozoa after 3 h of coincubation, and then further cultured for 7 h and fixed with acetic alcohol (acetic acid: methanol = 1:3, v/v) for at least 3 days (Kikuchi et al., 2002).

To assess the ability to develop to the blastocyst stage, the denuded oocytes were then in vitro cultured for 6 days in NCSU‐32 based media (IVC‐PyrLac for 2 days followed by IVC‐Glu for 4 days, Kikuchi et al., 2002) and fixed for assessment of blastocyst formation.

### 2.9 Assessment of fertilization and blastocyst formation

The fertilization status of the oocytes, including rates of sperm penetration, male pronucleus formation, and monospermy, was evaluated after staining with 1% aceto‐orcein. Development of oocytes after IVF to the blastocyst stage was evaluated after staining with 1% aceto‐orcein. An embryo with a clear blastocele was defined as a blastocyst, and the oocytes that remained at the mono‐cell stage or had fragmented were defined as degenerated oocytes or embryos.

### 2.10 Histological analysis

Ovaries obtained from neonatal pigs and entire fused grafts excised from the left kidneys were embedded in paraffin. For analysis of follicular status before grafting, 10 sections 6 μm thick were made from each neonatal ovary and stained with hematoxylin and eosin. All follicles showing oocyte nuclei were counted in each section and categorized as follows: primordial follicles with one layer of flattened granulosa cells, primary follicles with one layer of cuboidal granulosa cells, secondary follicles with two or more layers of granulosa cells but no antrum, and antral follicles with an antral cavity. Follicles were classified as atretic if they contained fragmented oocytes or granulosa cells with pyknotic nuclei and disrupted granulosa layers: these were excluded from data analysis. The data obtained from 10 sections from each ovary were pooled and the percentages were calculated. Mean (±*SEM*) percentages were calculated using the data obtained from three piglets, respectively: the values were expressed as those per piglet (*n* = 3). To assess follicular growth in ovarian xenografts, entire fused grafts were serially sectioned at 7 μm, and each 6th section was stained with hematoxylin and eosin. Diameters of all follicles showing oocyte nuclei were measured in each section, then these follicles were categorized as described earlier. The data obtained from every six sections were pooled and the percentages calculated. Mean (±*SEM)* number and percentages were calculated using the data obtained from three whole grafts, respectively: the values were expressed as those per mouse. Diameters of oocytes in antral follicles were also measured when the nucleolus of the oocyte was present.

### 2.11 Statistical analyses

All data were analyzed by one‐way ANOVA. When a significant effect was detected by ANOVA, the difference between two means was determined by Student's *t* test and differences among more than two means were determined by Tukey's test. The General Linear Models of Statistical Analysis Systems (9.2; SAS Inc., Cary, NC, USA) was used for this analysis. Differences at *p *<* *0.05 were considered to be significant.

## 3 RESULTS

### 3.1 Mice bearing Meishan ovarian tissue

Vaginal opening occurred in 43 of 48 mice (90%) that received Meishan ovarian tissue in experiments 1 to 3. The period between initial surgery and vaginal opening ranged from 31 to 50 days (37.7 ± 1.0 days, mean ± *SEM*,* n* = 43).

### 3.2 Growth of follicles and their oocytes in Meishan xenografts (Experiment 1)

**Figure 1:**
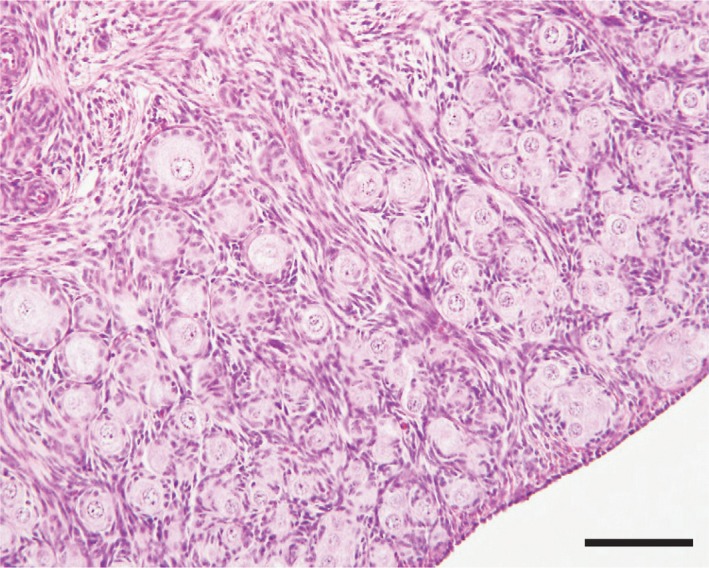
Histologic appearance of ovarian cortex area obtained from a Meishan pig aged 10 days. Scale bar represents 100 μm

**Figure 2:**
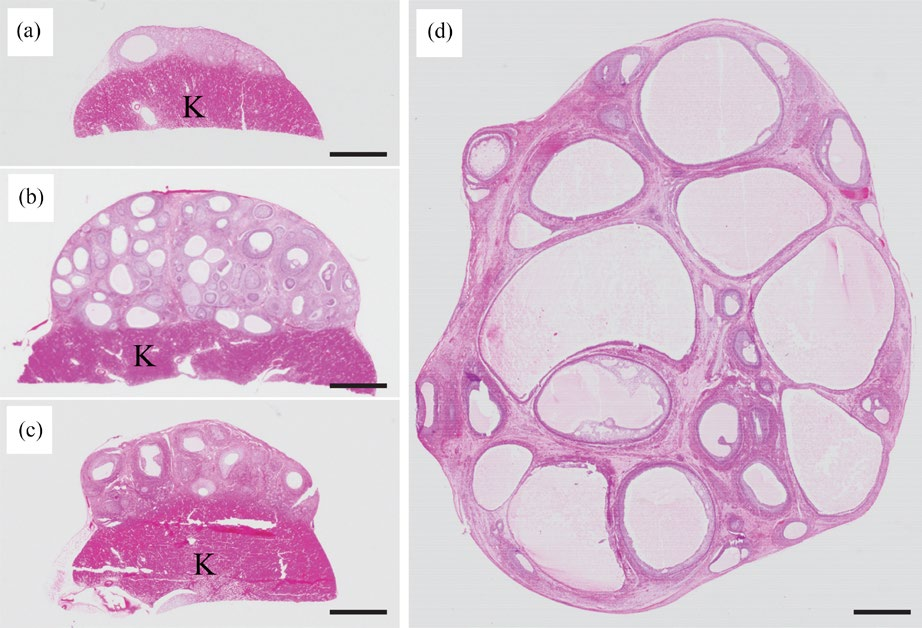
Histological appearance of Meishan ovarian grafts at low magnification in host mice of the (a) D10, (b) D60, (c) D10‐FSH, and (d) D60‐FSH groups. Ovarian grafts were examined 10 or 60 days after vaginal opening of host mice with no hormone treatment (D10 or D60 group). Grafts were also examined after infusion of porcine FSH for 13 days on day 10 or 60 after vaginal opening with a single injection of estradiol antiserum 7 days after the start of FSH infusion (D10‐FSH or D60‐FSH group). Letter K indicates kidney parenchyma. Scale bars represent 1 mm

**Figure 3:**
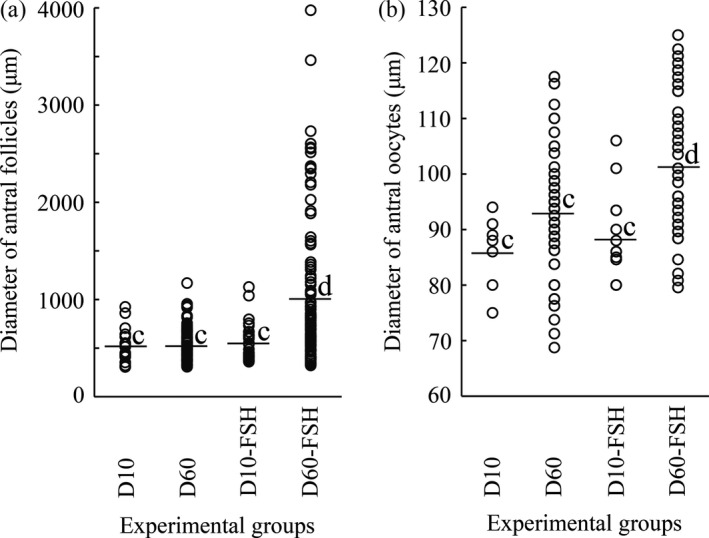
Distribution of the diameters of (a) antral follicles and (b) their oocytes in the D10, D60, D10‐FSH, and D60‐FSH groups. Diameters of antral follicles were measured when their oocyte (antral oocyte) contained a nucleus: those of antral oocytes showing a nucleolus were measured. Explanations about the experimental groups are given for Figure 2. Horizontal bars represent the mean values in each group. ^c–d^Values in each panel without common superscripts are significantly different (*p *<* *0.05)

**Figure 4:**
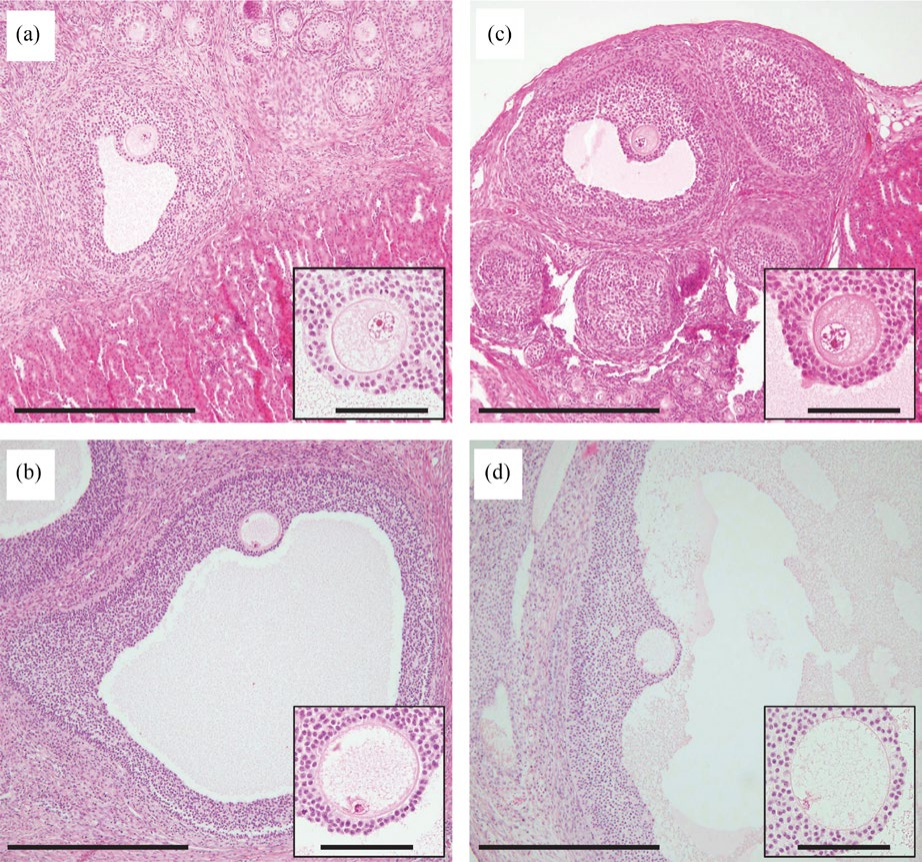
Representative relationships between the diameters of antral follicles and oocytes grown in Meishan xenografts in the (a) D10, (b) D60, (c) D10‐FSH, and (d) D60‐FSH groups. Explanations about the experimental groups are given for Figure 2. Image inserted into each panel shows a magnified view of the oocyte. Scale bars represent 500 μm in each panel or 100 μm in each insert

In the Meishan ovaries aged 9–14 days (before grafting), the primordial follicles accounted for 96% (96.1 ± 0.8, mean ±* SEM*,* n* = 3 piglets) of the total number of nonatretic follicles classified, and the remaining 4% comprised primary (3.5 ± 0.6%, *n* = 3) and secondary (0.4 ± 0.1%, *n* = 3) follicles: no antral follicles were present (Figure 1). Growth of nonatretic follicles in xenografts in each group is summarized in Table 1. In the D10 group, primordial follicles still accounted for the majority (81%) of nonatretic follicles in the xenografts, but a small number of follicles had progressed to the antral stage (Table 1 and Figure 2a). Xenografts in the D60 group contained many more antral follicles than those in the D10 group (*p *<* *0.05) (Table 1 and Figure 2b). Antra appeared when follicles reached 300–400 μm in the D10 and D60 groups (Figure 3a). Host mice in the two groups given FSH (D10‐ and D60‐FSH groups) showed no significant differences in the number or proportions of each follicle, when compared with those in the corresponding controls without FSH stimulation (D10 and D60 groups, respectively) (Table 1). However, the mean diameter of antral follicles in the D60‐FSH group was 998 ± 67 μm (±*SEM*,* n* = 116 follicles from three mice, range: 321–3,974 μm) (Figures 2d and 3a), being significantly (*p *<* *0.05) larger than that in the D60 group (514 ± 10 μm, *n* = 187, range: 308–1,168 μm) (Figures 2b and 3a). The mean diameter of antral follicles in the D10‐FSH group (547 ± 32 μm, *n* = 34, range: 359–1,128 μm) was not different from that in the D10 group (512 ± 42 μm, *n* = 18, range: 306–923 μm) (Figures 2a, 2c, and 3a). The diameters of oocysts in the antral follicles were 86 ± 2 μm (*n* = 7, range: 75–94 μm) in the D10, 93 ± 1 μm (*n* = 72, range: 69–118 μm) in the D60, 88 ± 2 μm (*n* = 14, range: 80–106 μm) in the D10‐FSH and 102 ± 2 μm (*n* = 51, range: 81–125 μm) in the D60‐FSH groups, respectively (Figures 3b and 4): the value in the D60‐FSH group was significantly (*p *<* *0.05) greater than in the other groups. When porcine oocytes were recovered from the right kidneys in three host mice, the total number of oocytes was 24, 19, and 71 in the D10‐, D30‐, and D60‐FSH groups, respectively: the number of large oocytes (≥115 μm) was 2, 8, and 15, respectively.

**Table 1:** Follicular development in Meishan ovarian grafts in host mice of the D10, D60, D10‐FSH, and D60‐FSH groups

Group^a^	Means^b^ ± *SEM* per mouse^c^				
	Primordial follicles	Primary follicles	Secondary follicles	Antral follicles	All follicles
D10	6,385 ± 2,257 (81.4 ± 2.8)	787 ± 171 (11.5 ± 1.9)	492 ± 122 (7.0 ± 0.9)	6 ± 2^d^ (0.1 ± 0.1^d^)	7,670 ± 2,532
D60	3,898 ± 915 (83.7 ± 0.6)	312 ± 70 (6.9 ± 0.9)	401 ± 132 (8.0 ± 1.2)	62 ± 16^e^ (1.3 ± 0.1^e^)	4,674 ± 1,123
D10‐FSH	7,300 ± 2,608 (84.6 ± 1.8)	730 ± 221 (9.1 ± 0.8)	459 ± 125 (6.0 ± 0.9)	11 ± 2^d^ (0.2 ± 0.1^d^)	8,499 ± 2,952
D60‐FSH	1,408 ± 303 (77.1 ± 2.2)	191 ± 24 (10.8 ± 0.7)	170 ± 18 (9.7 ± 0.8)	39 ± 6^e^ (2.4 ± 0.8^e^)	1,807 ± 340

^a^Mice in the D10 or D60 group received no hormone treatment and grafts were recovered on 10 or 60 days after vaginal opening. Mice in the D10‐ or D60‐FSH group received one osmotic pump containing porcine FSH (62.5 U/ml) for 13 days on 10 or 60 days after vaginal opening with a single injection of estradiol antiserum 7 days after the start of FSH infusion.

^b^Values are mean numbers (±*SEM*) of each categorized follicle showing no atretic signs followed by mean percentages (±*SEM*) (parenthesis).

^c^Entire fused grafts under the capsules of left kidneys of three mice were used for histological examination in each group.

^d‐e^Values in the same column without common superscripts are significantly different (*p *<* *0.05).

### 3.3 Maturation ability of recovered oocytes (Experiment 2)

**Figure 5:**
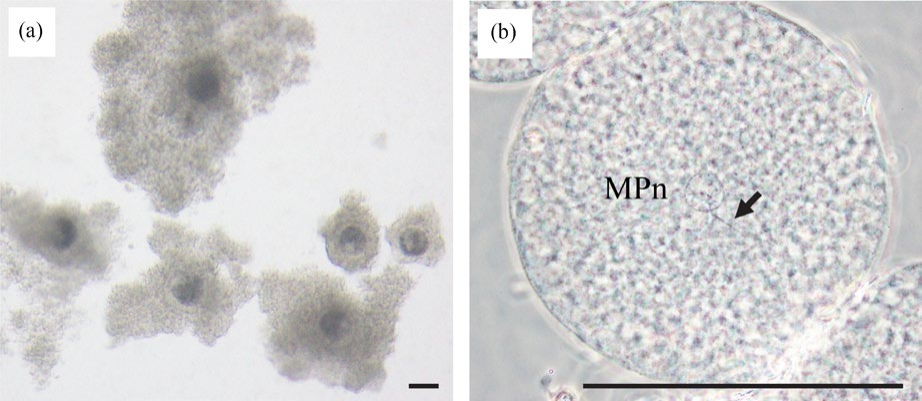
(a) Cumulus‐oocyte complexes and (b) in vitro fertilization of oocytes recovered from host mice. Host mice received one osmotic pump containing 125 U/ml porcine FSH for 13 days on day 60 after vaginal opening with a single injection of estradiol antiserum 7 days after the start of FSH infusion. The arrow indicates a sperm tail associated with a male pronucleus. The female pronucleus is in a different focal plane. Bars indicate 100 μm. MPn, male pronucleus

There was no significant (*p *<* *0.1) difference in the total number of oocytes between the FSH‐62.5 and FSH‐125 groups (Table 2). However, a greater (*p *<* *0.05) number of large oocytes (≥115 μm) or oocytes with maturation ability was obtained from host mice of the FSH‐125 group, as compared to the FSH‐62.5 group. Many of the oocytes isolated from the FSH‐62.5 and FSH‐125 mice were surrounded by cumulus cells (Figure 5a). The ratios of metaphase II oocytes to the number of large oocytes (≥115 μm) subjected to IVM (maturation rate) was 26% in the FSH‐62.5 group and 33% in the FSH‐125 group.

**Table 2:** Number and meiotic competence of Meishan oocytes recovered from host mice given two doses of FSH

Group^a^	No. of mice	Total No. of oocytes recovered^b^	No. of oocytes larger than 115 μm^c^	No. of mature oocytes^d^
FSH‐62.5	6	39.3 ± 10.3 (236)	17.8 ± 4.4^e^ (107)	4.7 ± 1.5^e^ (28)
FSH‐125	6	56.8 ± 4.1 (341)	34.5 ± 4.4^f^ (207)	11.3 ± 1.7^f^ (68)

^a^ Host mice received one osmotic pump containing porcine FSH (62.5 or 125 U/ml) for 13 days on 60 days after vaginal opening with a single injection of estradiol antiserum 7 days after the beginning of FSH infusion.

^b‐d^The number of oocytes in each category is represented as the mean ± *SEM* per mouse followed by the total number (in parentheses).

^e‐f^Values in the same column without common superscripts are significantly different (*p *<* *0.05).

### 3.4 Fertilizing and developmental ability of recovered oocytes (Experiment 3)

Thirty‐eight matured oocytes obtained from five host mice were subjected to IVF using Meishan spermatozoa. The percentage of oocytes penetrated by at least one spermatozoon, calculated as the number of oocytes with sperm penetration/number of mature oocytes, was 61% (23/38). The ratio of oocytes with a male pronucleus (or pronuclei) (MPn) to the number of sperm‐penetrated oocytes (MPn formation rate) was 100% (23/23) (Figure 5b). The ratio of oocytes showing monospermy, as the number of monospermic oocytes/number of sperm‐penetrated oocytes, was 17% (4/23). Sixty‐six matured oocytes obtained from eight host mice were subjected to IVF then further cultured for 6 days, but no oocytes developed to the blastocyst stage.

## 4 DISCUSSION

Indigenous pigs are expected to be a reservoir of unique genetic diversity; however, their population has been decreasing in several regions of the world due to the introduction of Western commercial breeds or civil wars (Baumung & Wieczorek, 2015). Xenografting of ovarian tissue might be an aid to conservation of genetic diversity of indigenous pigs, since it can provide opportunities for generation of mature oocytes from neonatal and recently deceased females, in addition to conventional reproductive methods collecting oocytes from sexually mature females. In order to utilize neonatal oocytes from indigenous pigs, it is necessary to know whether their ovarian tissue can acquire the capacity to produce fully grown oocytes by xenografting, and whether these xenogeneic oocytes have developmental competence. The findings of this study confirmed that ovarian tissue obtained from indigenous Meishan piglets can produce oocytes with in‐vitro fertilization ability, although no blastocysts were obtained from these oocytes after IVC.

In Meishan ovaries aged 7–14 days (before grafting), the proportions of primordial, primary, and secondary follicles were 96.1%, 3.5%, and 0.4%, respectively. These proportions were similar to those in Meishan neonatal ovaries obtained in a previous study (Miyano et al., 1990), and not different from those in 20‐day‐old Western breed ovaries used in our previous xenografting study (Kaneko et al., 2003). After grafting of Meishan ovarian tissue, host mice exhibited vaginal opening on 38 (±1.0) days after grafting surgery, accompanied by formation of a small number of antral follicles. The present period between the surgery and vaginal opening or formation of antral follicles was shorter than that for mice receiving grafts from 20‐day‐old Western piglets using the same procedures (63 ± 2.5 days, Kaneko et al., 2003). These findings are consistent with previous studies indicating that Meishan gilts (McCoard et al., 2003; Miyano et al., 1990) develop antral follicles 1 month earlier than Western breeds (Oxender et al., 1979), and thereby reach puberty earlier than Western breeds (Bazer et al., 1988; Faillace et al., 1994; White et al., 1993). Thus, even in the same milieu (i.e., nude mice), Meishan ovarian xenografts showed more rapid growth than Western‐breed grafts, as well as Meishan testicular xenografts (Kaneko, Kikuchi1, Men, & Noguchi, 2019): a characteristic that appears to be inherent to Meishan gonads.

Histological examination revealed that the number of small antral follicles without atretic signs increased considerably in Meishan xenografts from days 10 to 60 after grafting surgery. Ovarian xenografts in the D10 and D60 groups correspond to ovaries in Meishan gilts aged at 43 and 93 days, respectively. Promotion of antral follicle formation similarly occurs in Meishan gilts between 45 and 90 days after birth (Miyano et al., 1990). Infusion of porcine FSH started on day 60 induced enlargement of antral follicles (D60‐FSH group), but the same treatment started on day 10 had no effect on the size of antral follicles (D10‐FSH group). These results suggest that small antral follicles present in D60‐xenografts are more sensitive to exogenous FSH than those in the D10‐xenografts. This hypothesis is supported by previous findings (Miyano et al., 1990) that treatment with eCG consistently promoted the formation of large antral follicles in Meishan gilts aged 75 days, whereas the same treatment rarely did so in gilts aged between 45 and 60 days, despite the presence of small antral follicles. In parallel with the status of antral follicle formation, xenografts in the D60 group contained many more oocytes in antral follicles, although the mean diameter of these oocytes, determined histologically, was not different from that in the D10 group. Administration of FSH to host mice on Day 60 induced an upper shift in the distribution of oocyte diameters, and several oocytes reached 115 μm in diameter (presumably with maturational ability) in the D60‐FSH group. Moreover, the number of large oocytes (≥115 μm in diameter) recovered from host mice was 15 in the D60‐FSH group, whereas it was 2 in the D10‐FSH and 8 in the D30‐FSH groups. It seems likely that Meishan xenografts require such a long period (60 days) before FSH treatment to attain good performance in terms of follicular growth and oocyte recovery, like ovarian xenografts from Western breeds (Kaneko et al., 2003, 2006).

We further examined the dose‐effects of FSH on the meiotic competence of oocytes in the grafted tissue. Many oocytes recovered from the FSH‐62.5 and FSH‐125 groups were surrounded by abundant cumulus cells. However, a higher dosage of FSH increased the number of large oocytes (≥115 μm) recovered and oocytes with in vitro maturation ability, compared to those after a lower FSH dosage. The results indicate that FSH treatment with a dose of 125 U/ml had beneficial effects on the meiotic activity of oocytes in the grafted tissue from neonatal Meishan pigs.

In the FSH‐125 group, 33% of large oocytes (≥115 μm) matured in vitro. This ratio is lower than in our previous results (70%) after IVM of oocytes collected from prepubertal gilts using the same IVM system (Kikuchi et al., 1999, 2002; Nakamura, Tajima, & Kikuchi, 2017): this low maturation ability seems to be common for porcine xenogeneic oocytes (Kaneko et al., 2003, 2006, 2016), but it was still enough to be useful. We demonstrated that mature oocytes from the FSH‐125 group were fertilized (61% of mature oocytes) in our IVF system (Kikuchi et al., 2002), as shown by the formation of female and male pronuclei. These results indicate that Meishan oocytes, even grown in host mice, can acquire in vitro fertilization ability. However, no blastocyst was able to develop from these IVF oocytes. Oocytes recovered from Western‐breed grafts also show difficulty in reaching the blastocyst stage (Kaneko et al., 2006), but fusion of cytoplasm prepared from oocytes obtained from neonatal gilts that have been proven to reach the blastocyst stage improved the ability of xenogeneic oocytes to form blastocysts (Kaneko et al., 2013). This suggests that xenogeneic oocytes lack cytoplasmic factors which are necessary for embryonic development (so called cytoplasmic maturation). Microarray analyses have revealed that genes involved in metabolism, lipid metabolism, and gonadal development are downregulated in rat ovarian tissue grafted into immunodeficient mice (Agca, Lucy, & Agca, 2009). Considering that substances such as maternal RNA, proteins, and cyclic adenosine monophosphate accumulate in ooplasm and affect embryonic development (Fair et al., 2004; Gandolfi & Gandolfi, 2001), xenogeneic oocytes of Meishan pigs have difficulty in achieving full cytoplasmic maturation to support embryonic development. It might be necessary to directly replace the cytoplasm of xenogeneic oocytes with intact cytoplasm by nuclear transfer. Transfer of a nucleus from a poor‐quality oocyte at the germinal vesicle or metaphase II stage into an enucleated oocyte with full developmental competence has been reported to produce embryos in mice (Bai, Liu, & Wang, 2006; Liu, Van Der Elst, & Dhont, 2003; Mitsui, Yoshizawa, Matsumoto, & Fukui, 2009) and cattle (Bao et al., 2003).

## 5 CONCLUSION

We have been able to obtain large oocytes (≥115 μm in diameter) from Meishan neonatal ovarian tissue grafted into nude mice. Subsequent IVM and IVF resulted in successful production of fertilized oocytes. This approach may have potential utilization for preservation of genetic resources in indigenous pigs.
